# Case Report: PD-1 Blockade Combined Autologous Hematopoietic Stem Cell Transplantation With Modified BEAM Regimen Containing High-Dose Cytarabine to Treat R/R Hodgkin's Lymphoma

**DOI:** 10.3389/fmed.2021.693023

**Published:** 2021-07-07

**Authors:** Xiaoqi Wang, Kaniel Cassady, Zhongmin Zou, Xi Zhang, Yimei Feng

**Affiliations:** ^1^Medical Center of Hematology, The Xinqiao Hospital of Third Military Medical University, Chongqing, China; ^2^Irell and Manella Graduate School of Biological Sciences of City of Hope, Duarte, CA, United States; ^3^Department of Chemical Defense Medicine, School of Military Preventive Medicine, Third Military Medical University, Chongqing, China

**Keywords:** PD1, autologous stem cell transplantation, Hodgkin's lymphoma, beam, case report

## Abstract

The emergence of new drugs has provided additional options in the treatment of relapsed and refractory (R/R) Hodgkin's lymphoma (HL). However, the use of autologous stem cell transplantation (ASCT) has not been completely replaced in this setting. The use of anti-programmed death-1 (PD-1) antibody bridging to ASCT and as maintenance after transplantation is a novel approach in HL treatment. In this case, we report that PD-1 monoclonal antibody (mAb) plus ASCT with modified BEAM regimen (carmustine + etoposide + cytarabine + melphalan) containing high-dose cytarabine to treat R/R HL may represent a promising regimen in this difficult-to-treat setting.

## Introduction

Hodgkin's lymphoma (HL) is a malignant proliferative tumor derived from the lymphatic system, with the number of newly diagnosed HL patients worldwide reaching 79,990 cases in 2018 and accounting for 13.6% of all lymphomas (1). More than 80% of HL patients achieve remission and long-term disease remission after standard first-line chemotherapy, radiotherapy, and radiotherapy plus chemotherapy, but disease in ~5–10% of HL patients does not respond to initial treatment, while disease in ~10–30% of HL patients progresses or relapses after achieving complete remission (CR) (2) by first-line treatment. Among these patients, recurrence rate is higher in patients with intermediate and advanced stage, and overall prognosis is worse for first-onset refractory and relapsed cases within 1 year of follow-up (3). Therefore, relapsed and refractory (R/R) HL is still a clinical challenge and area of research interest.

To date, autologous stem cell transplantation (ASCT) is a mainstay in the treatment paradigm for R/R HL (4). The emergence of new drugs [such as CD30 monoclonal antibody (mAb), programmed death-1 (PD-1)/programmed death-ligand 1 (PD-L1) antibody, etc.] has provided additional options in the treatment of R/R HL; however, these regimens have not yet displaced the use of ASCT in these patients (5). The BEAM regimen (carmustine + etoposide + cytarabine + melphalan) as a conditioning scheme in ASCT has proven to be the most effective regimen for HL (6). However, other reports suggest that the BEAM regimen can be optimized, for instance, by using a larger dose of cytarabine, for greater efficacy in lymphoma histologies such as mantle cell lymphoma (MCL) and others (7, 8). Herein, we provide the first reported case of a patient with HL achieving CR treated with PD-1 antibody backbone therapy while undergoing ASCT following a modified BEAM conditioning regimen. This is also the first report of a modified BEAM regimen as a conditioning regimen, with large doses of cytarabine, in this setting. Together, this regimen may provide an additional option in difficult-to-treat R/R HL patients.

## Case Presentation

A 28-year-old male was admitted to the hospital in October 2017 due to a complaint of “a lump in the left cervical lymph node.” Related examinations were conducted. Routine blood examination showed white blood cell count (WBC) 12.0 ×10^9^/L, hemoglobin (HGB) 119 g/L, and platelet count (PLT) 429 ×10^9^/L. The albumin level was 39.1 g/L. The neck lymph node biopsy and pathological staining showed tumor cells with CD30^+^, CD15^+^, PAX-5^+^, and LCA^−^ and background cells with CD20 few+, CD3 majority+, CD43^+^, S100 scattered+, and CD1a scattered+. PET/CT examination showed multiple swollen lymph nodes throughout the body (bilateral neck, clavicle area, mediastinum, axilla, diaphragm, peritoneum, and splenic hilum), the maximum standard uptake value (SUV) was 12.4; spleen and multiple sites of bones were invaded by tumors. No lymphoma cells were founded in the sample from bone marrow aspiration. At last, the diagnosis of this patient was classic HL [IV B phase, International Prognostic Score (IPS) score 4].

**Figure 1:**
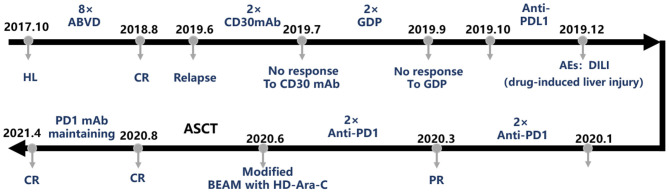
The treatment course of this case. ABVD, doxorubicin + bleomycin + vinblastine + dacarbazine; AE, adverse event; ASCT, autologous stem cell transplantation; BEAM, carmustine + etoposide + cytarabine + melphalan; CR, complete remission; HL, Hodgkin's lymphoma; GDP, gemcitabine + cisplatin + dexamethasone; mAb, monoclonal antibody; PD-1, programmed death-1; PD-L1, programmed death-ligand 1; PR, partial remission.

Subsequently, the patient received ABVD chemotherapy regimen (doxorubicin liposome 40 mg at day 1 and day 14, bleomycin 1.5 wU at day 1 and day 14, vinblastine 4 mg at day 1 and day 14, dacarbazine 600 mg at day 1 and day 14) according to the HL treatment guidelines. Then, the patient was advised to receive a PET/CT examination after two cycles of ABVD according to National Comprehensive Cancer Network (NCCN) guidelines but had concerns about receiving PET/CT again in a short time and only consented to an ultrasound and chest CT examination. The results showed that the previously enlarged lymph nodes in the neck, mediastinum, armpit, and groin shrank significantly. After the fourth course of ABVD, the patient consented to receive PET/CT, and it showed CR. After continuing four cycles of ABVD regimen treatment (eight cycles of ABVD in total), the patient was followed by regular clinic follow-up (Figure 1).

In October 2018, PET/CT examination revealed multiple enlarged lymph nodes throughout the body, and SUVmax was 8.95. Lymphoma recurrence was suspected. Although the left clavicle lymph node biopsy revealed fibrous collagen tissue hyperplasia with glass-like changes, no tumor cells were seen. Follow-up was continued until the end of May 2019, wherein the patient developed a recurrent cervical lymphadenopathy, accompanied by low fever and night sweats. PET/CT examination at the beginning of June showed that there were multiple lymph nodes with swelling throughout the body, and the number of enlarged lymph nodes had increased significantly, and SUVmax was 10.06, with tumor cell invasion in the spleen and multiple bones. This time, biopsy of the right cervical lymph node revealed classic HL. Bone marrow biopsy results showed no lymphoma cell invasion. The recurrence of HL was diagnosed. Therefore, the patient was admitted to the hospital again for further management.

According to NCCN guidelines (9) for the use of CD30 mAb in the treatment of HL (10, 11), two doses of CD30 mAb [brentuximab vedotin (BV)] were injected in June and July 2019. During follow-up, the patient stated that the size of the cervical lymph nodes shrunk, and fever and night sweats were alleviated. However, PET/CT examination in August revealed that the volume of multiple lymph nodes all over the body were not significantly reduced compared with previous results, although the SUV values of multiple lesions and bones were decreased. Deauville score was 5. Bone marrow biopsy results revealed that typical R-S cells were found in biopsy. Therefore, we considered that lymphoma had invaded the patient's bone marrow and was not well-treated by CD30 mAb. Thus, we considered additional candidate therapies for this patient.

In August and September 2019, the patient was treated with second-line chemotherapy regimen, two cycles of GDP chemotherapy (gemcitabine 1.6 g at day 1, cisplatin 40 mg for days 1–3, dexamethasone 40 mg for days 1–4). The PET/CT in October still showed multiple lymphadenopathies throughout the body. There were new lesions in the esophagus, bilateral iliac vessels, and the right inguinal area. Lymphoma cell infiltration was discovered in multiple bones throughout the body to a greater extent than initially diagnosed. Deauville score was 5 points. These results demonstrated the ineffectiveness of the second-line chemotherapy.

Subsequently, the patient volunteered and was enrolled in the “Phase II clinical study on the efficacy and safety of TQB2450 (PD-L1 mAb) in the treatment of relapsed/refractory classic Hodgkin's lymphoma (clinical trial number: CTR20190097)” on October 24, 2019. After the first PD-L1 mAb treatment, the patient's liver function test displayed an obvious increase in transaminase, suggesting the occurrence of liver damage. Subsequently, the patient was given hepatoprotective treatment and withdrew from the clinical trial. In December, there were still multiple swollen lymph nodes in the PET/CT results. The Deauville score was 4 points. However, there were new lesions in the pelvic cavity behind the peritoneum, indicating poor response to the treatment. Again, additional options to treat this patient were assessed, and PD-1 therapy was considered.

The patient was administered two doses of carrelizumab (anti-PD-1 mAb) in January and February 2020, and PET/CT examination showed that the size of the retroperitoneal and pelvic lymph nodes was smaller, but new lesions occurred in the left clavicular lymph nodes. The Deauville score was 4 points. Bone marrow biopsy did not reveal tumor cells. Thus, treatment with PD-1 mAb led to a partial remission (PR) in this patient. At this stage, according to treatment guidelines and the willingness of the patient, we initiated the procedure of ASCT. The granulocyte colony-stimulating factor (G-CSF) procedure to mobilize peripheral blood stem cells was performed on March 9, 2020, and peripheral blood stem cells were collected on March 13 and 14. Mononuclear cell (MNC) level was 10.09 ×10^8^/kg, and CD34^+^ cell level was 2.44 ×10^6^/kg. In April and May 2020, the patient received another two doses of the anti-PD-1 mAb carrelizumab. Ultrasound examination before transplantation indicated that lymph nodes had shrunk.

**Figure 2:**
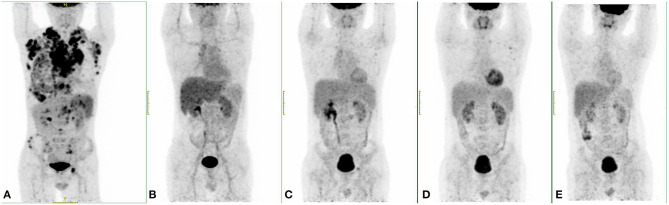
PET/CT results of this case at different time points. **(A)** The PET/CT image when diagnosed. **(B)** A PET/CT examination after the fourth cycle of ABVD (doxorubicin, bleomycin, vinblastine, dacarbazine) showed complete remission (CR). **(C)** After programmed death-ligand 1 (PD-L1) monoclonal antibody (mAb) treatment, there were still multiple swollen lymph nodes in the PET/CT results, and new lesions were found in the pelvic cavity behind the peritoneum. **(D)** After two doses of programmed death-1 (PD-1) mAb treatment, PET/CT examination showed that the size of the retroperitoneal and pelvic lymph nodes was smaller, but new lesions occurred in the left clavicular lymph nodes. **(E)** After autologous stem cell transplantation (ASCT), this patient reached CR.

The patient entered the laminar flow chamber on June 18, 2020, and was treated with modified BEAM regimen (carmustine 500 m at -day 6, etoposide 0.1 g/m^2^ for -day 6~-day 3, cytarabine 1 g/m^2^/day for -day 6~-day 3, dexamethasone 10 mg for -day 6~-day 2, melphalan 250 mg at -day 2). Autologous peripheral blood stem cell infusion was conducted on June 24 and 25. Routine blood examination after 12 days of transplantation indicated hematopoietic reconstitution with WBC 8.06 ×10^9^/L, HGB 124 g/L, and PLT 30 ×10^9^/L. PET/CT examination in August 2020 indicated that the patient achieved CR (Figure 2). The patient was treated once more with carrelizumab and discharged from the hospital on September 9, 2020. At present, the patient continues to be followed up regularly with CR status and receives a maintenance dose of PD-1 mAb every 3 months.

## Discussion and Literature Review

Here, we report a case of a patient with typical R/R HL, which did not respond when treated with ABVD, GDP chemotherapy regimens, and emerging therapeutic options (BV and PD-L1 mAb). Administration of PD-1 mAb as a backbone to a BEAM modified (high-dose cytarabine) conditioning regimen prior to ASCT resulted in CR in this patient.

At present, promising new drugs for HL have improved outcomes in difficult-to-treat patients. BV mAb targets the CD30 molecule and carries the anti-tubulin drug monomethyl auristatin E (MMAE) to form a new antibody–drug conjugate (ADC) that binds to CD30 antigen and exerts MMAE tumor killing function (11). For patients who failed auto-hematopoietic stem cell transplantation (HSCT) treatment, the overall response rate and CR rate of BV monotherapy reached 75 and 33%, respectively. The median value of progression-free survival (PFS) time of patients who reached CR was 9.3 months, medium value of the duration of response (DOR) time was 20.5 months. The 5-year overall survival (OS) rate and PFS rate were 41 and 22%, respectively (12, 13). On May 2020, the China Food and Drug Administration (CFDA) officially approved BV for the treatment of adult patients with CD30-positive relapsed or refractory systemic anaplastic large cell lymphoma (sALCL) and classic Hodgkin's lymphoma (cHL). But in this case, PET/CT results of the patient showed no significant improvement after using two doses of BV treatment, and HL tumor cells invaded bone marrow, which forced us to reconsider the treatment strategy.

PD-1/PD-L1 immune checkpoints associated with T-cell exhaustion have attracted attention recently in tumor treatment (14, 15). After one dose of PD-L1-blocking mAb was given to our patient, his PET/CT score decreased from 5 to 4 points, but with no obvious symptom relief. At the same time, there were new lesions in the retroperitoneum and pelvis. Additionally, severe adverse events (AEs) occurred during the treatment, resulting in high liver transaminase. Therefore, the patient withdrew from the clinical trial. Subsequently, he was treated with PD-1 mAb. The efficacy of PD-1 in treating HL in real world has been encouraging (16). For example, in one study, 53 cases of R/R HL were enrolled, among them, 68% is objective response rate (ORR) (45% CR, 23% PR), 12M-OS 89%, 12M-PFS 75%, median value for PFS is 29 M. In our patient, after receiving four doses of PD-1-blocking mAb, ultrasound revealed that the size of the lymph nodes had shrunk, and disease was in PR, likely facilitating tumor burden reduction prior to ASCT. Both PD-1 and PD-L1 antibodies function as an “immune brake” with similar yet distinct mechanisms, as PD-1 blockade primarily acts directly on target T cells, while PD-L1 blockade mediates its effect indirectly on T cells *via* antigen presenting cell (APC) or tumor cell targeting. At present, PD-L1 mAb has no indications for hematological tumors, and it is still undergoing clinical trials. There are no direct references in comparison with the efficacy of PD-1 and PD-L1. In this case, we used PD-1 blockade as a backbone pre- and post-ASCT. The impact of PD-1 in this case cannot be overlooked; however, further clinical evidence is needed to distinguish the impact of PD-1 vs. PD-L1 in this setting, as well as the differential contribution of PD-1 vs. modified BEAM regimen in this setting.

Related to the conditioning regimen, two randomized controlled phase III clinical studies found that compared with conventional chemotherapy, salvage high-dose chemotherapy combined with autologous HSCT (HDCT/auto-HSCT) can significantly increase the disease-free survival (DFS) of R/R HL patients. These findings laid the foundation of HDCT/auto-HSCT as a standard treatment plan for R/R patients (17, 18). BEAM regimen is reportedly recognized as the best preconditioning regimen for HL. The overall survival rate of the BEAM pretreatment regimen for HL patients is the highest with a 3-year PFS of 62% and a 3-year OS of 79% (6). In this case, we added the dose of cytarabine in the BEAM regimen. The Ara-C of conventional BEAM is 200 mg/m^2^, but we use 1,000 mg/m^2^ based on the following clinical evidence. The effect of medium and large doses of Ara-C in lymphoma has been reported (Table 1). A report showed that medium and large doses of cytarabine can improve the efficacy of MCL treatment (23). In that study, patients were divided by two groups: Group A: 6 RCHOP with (pretreatment: TBI + Cy) ASCT; Group B: 3 RCHOP with 3 DHAP (dexamethasone, high-dose cytarabine, oxaliplatin) and (pretreatment: TBI, high-dose Ara-C, Mel) ASCT. The CR rate and CR/unconfirmed complete response (CRu) combination rate of group B were significantly higher than those of group A (26 vs. 39%, *p* = 0.012 and 41 vs. 60%, *p* = 0.0003). After an average of 27 months' follow-up, the time to treatment failure (TTF) of group B patients was significantly prolonged (49 months vs. no response (NR); *p* = 0.0384). Hence, the study indicated that high-dose cytarabine plus ASCT can significantly increase the CR rate and TTF without clinically relevant toxicity increase (23). Additionally, in the chemotherapy regimen for R/R lymphoma, the DHAP regimen containing high-dose cytarabine is more efficient compared to non-Hodgkin's lymphoma (NHL) high-dose cytarabine, with both higher OS and PFS, thus increasing the efficacy of lymphoma (HL) treatment (7). Merryman et al. (19) recently reported that PD-1 mAb bridged ASCT with conventional BEAM to treat R/R HL can make 18-month PFS reach 78%. Therefore, in the treatment of this patient, we reasonably combined the advantages of PD-1 and ASCT. Meanwhile, we increased the dose of Ara-C in BEAM conditioning regimen, which is also the most innovative feature of this case. As a result, this patient benefited from PD-1 bridging ASCT strategy (Table 1). Previous publications from our center suggest that the use of ASCT as a first-line consolidation treatment could improve patients' outcome with advanced-stage high-risk HL, whose interim PET/CT was positive (4). For high-risk HL patients, we recommend ASCT be considered a frontline therapy.

**Table 1:** Reports of high-dose cytarabine combined with ASCT in the treatment of blood diseases.

**Author (References)**	**Case**	**Disease**	**Pre-HSCT chemotherapy**	**Ara-C dose before ASCT**	**Conditioning regimen in ASCT**	**Ara-C dose in ASCT**	**Outcome**
Current Study	1	HL	PD-1 antibody	/	Modified BEAM	1,000 mg/m^2^	CR and survived
Merryman et al. (19)	53	HL	PD-1 antibody	/	BEAM	200 mg/m^2^	18-month PFS 78%
Van't Veer et al. (20)	87	MCL	RTX + HD-Ara-C	2,000 mg/m^2^	BEAM	200 mg/m^2^	4-year PFS 46% 4-year OS 79%
Rigacci et al. (7)	70	DLBCL,HL	Oxaliplatin + Ara-C + Dex	2,000 mg/m^2^	Not mentioned	/	2-year PFS 44% 2-year OS 71%
Hermine et al. (21)	232	MCL	RCHOP + DHAP	2,000 mg/m^2^	TBI + Cytarabine + Melphalan	1.5 g/m^2^	5-year OS 65%
Aoki et al. (22)	4	1 MDS,3 AML	Not mentioned	/	Ara-C + fludarabine + cyclophosphamide	2–3 g/m^2^	Survived

*HL, Hodgkin's lymphoma; MCL, mantle cell lymphoma; DLBCL, diffuse large B-cell lymphoma; MDS, myelodysplastic syndrome; AML, acute myeloid leukemia; RTX, rituximab; Dex, dexamethasone; Ara-C, cytarabine; CR, complete remission; PFS, progression-free survival; OS, overall survival; DHAP, dexamethasone; high-dose cytarabine, oxaliplatin*.

## Conclusion

The present case report further suggests that PD-1 mAb use as a backbone to ASCT with modified BEAM containing high-dose cytarabine to treat R/R HL may represent a promising regimen in this difficult-to-treat setting and is the first report of such a strategy to our knowledge. It is likely that PD-1 mAb played a dominant role in this regimen; however, additional clinical evidence is needed to further evaluate the contribution of components in this regimen and setting.

## Data Availability Statement

The raw data supporting the conclusions of this article will be made available by the authors, without undue reservation.

## Ethics Statement

The studies involving human participants were reviewed and approved by Xinqiao Hospital Ethics Committee. The patients/participants provided their written informed consent to participate in this study. Written informed consent was obtained from the individual(s) for the publication of any potentially identifiable images or data included in this article.

## Author Contributions

YF contributed to the design and conceptualization of the research, design of data analyses, interpretation of data, collection of data, and writing of the manuscript. XW, KC, ZZ, and XZ edited this report. XZ and YF funded the work. All authors contributed to the article and approved the submitted version.

## Conflict of Interest

The authors declare that the research was conducted in the absence of any commercial or financial relationships that could be construed as a potential conflict of interest.
